# Neuropsychological and psychosocial assessment of small and non-small lung cancer patients: a study protocol

**DOI:** 10.3389/fpsyg.2025.1502793

**Published:** 2025-04-30

**Authors:** Benedetta Capetti, Lorenzo Conti, Chiara Marzorati, Vincenzo Bagnardi, Matteo Chiari, Monica Casiraghi, Roberto Grasso, Gabriella Pravettoni

**Affiliations:** ^1^Applied Research Division for Cognitive and Psychological Science, European Institute of Oncology IRCCS, Milan, Italy; ^2^Department of Oncology and Haemato-Oncology, University of Milan, Milan, Italy; ^3^Department of Statistics and Quantitative Methods, University of Milan-Bicocca, Milan, Italy; ^4^Division of Thoracic Surgery, European Institute of Oncology IRCCS, Milan, Italy

**Keywords:** lung cancer, cancer-related cognitive impairment, longitudinal study, neuropsychological assessment, neurotoxicity

## Abstract

Early diagnosis and effective treatments have favored the survival of cancer patients but have also generated adverse consequences including cognitive impairment and psychological distress, which are related to both disease progression and the side effects of pharmacological agents. However, there is little data in the literature concerning such adverse effects in patients with lung cancer. Here, we describe the protocol of a study aiming to investigate the occurrence of cognitive impairment in patients with non-small-cell lung cancer and small-cell lung cancer undergoing adjuvant therapies or surgery in the year following enrollment. This longitudinal study will recruit around 200 lung cancer patients. To explore the cognitive profile pre- and post-oncological treatment, a cognitive evaluation will be administered to each lung cancer patient at baseline (T0), 4 (T1), and 12 months (T2) after the end of treatments. A cognitive screening will be assessed with the Montreal Cognitive Assessment and Mini-Mental State Examination. Executive functions will be investigated with the Frontal Assessment Battery, the Stroop Color Word test and the phonemic fluency test. Memory and learning will be examined with Rey's auditory verbal learning test, whereas working memory will be assessed with the Digit Span test and the Corsi Block-tapping Test. Finally, attention will be investigated with the Trail Making Test and the Symbol Digit Modalities Test. In addition, perceived cognitive impairment, anxious and depressive symptoms, cognitive reserve, sleep patterns, and patient's quality of life will be also investigated using self-report tools. The cognitive impairment will be identified by adopting the criteria proposed by the International Cognition and Cancer Task Force. This trial received approval from the ethical committee of the Institutes of Scientific Research and Healthcare, IRCCS, European Institute of Oncology (UID_IEO 2027). The results could have relevant implications for managing cognitive impairment and its impact on the quality of life of lung cancer patients. Through a systematic cognitive assessment and its associated risk factors, this study aims to provide valuable insights into clinical practice, enhancing the development of neuropsychological protocols.

## 1 Background

The improvements in early cancer detection and efficacy of innovative treatments developed in recent years have supported a prolonged lifespan of cancer patients, generating however the onset of long-term consequences. Cancer survivors must manage different clinical needs including cognitive impairment and psychological distress, which are related to both disease progression and side effects of pharmacological agents (Di Iulio et al., 2019; Lange et al., 2019).

Indeed, an increasing number of neuropsychological studies show that individuals with non-central nervous system (non-CNS) cancers might experience cognitive difficulties, with detrimental effects on social and emotional wellbeing and negative consequences on patient's quality of life (QoL; **?**Wefel et al., 2015; Joly et al., 2015; Mayo et al., 2021). Cognitive changes in cancer patients are frequently related to a reduction in work engagement, unemployment, and disruption in the ability to be engaged in routine activities; even minor perceived impairments have been associated with significant impacts on daily functioning and QoL (Chao et al., 2021).

A multitude of factors and treatments seem to be associated with cognitive dysfunctions in cancer patients. Firstly, cognitive symptoms were reported mainly after chemotherapies, but also other cancer treatments (e.g., immunotherapies, endocrine therapies, and cancer surgery) contribute to the onset of clinical difficulties such as reduced concentration, fatigue, and mood alterations (Di Iulio et al., 2019; Lange et al., 2019; Wefel et al., 2015; Mayo et al., 2021; Wefel et al., 2011; **?**; Joly et al., 2015). Moreover, cognitive impairment has been found even before the start of systemic treatments for non-CNS cancers (Joly et al., 2015; Mayo et al., 2021; Lange and Joly, 2017). Finally, it has been shown that the cognitive aspects of patients can influence treatment effectiveness, compliance, and overall patient satisfaction (Cutica et al., 2014).

For these reasons, cognitive dysfunctions were classified as a unique term “cancer-related cognitive impairment” (CRCI), affecting ~30%−40% of non-CNS-cancer patients (Di Iulio et al., 2019; Mayo et al., 2021; Janelsins et al., 2014; Dos Santos et al., 2020). CRCI mainly involves an impairment of multiple cognitive domains, including short-term memory, working memory, attention, executive functions, and information processing speed (Di Iulio et al., 2019; Dos Santos et al., 2020). These cognitive alterations are also supported by neuroimaging studies that have shown brain changes associated with this cognitive profile (Lange et al., 2019; Conti et al., 2024).

Besides exposure to cytotoxic drugs, several factors are involved in CRCI and represent a higher risk of developing cognitive dysfunction, including psychological and sociodemographic variables, lifestyle factors, and genetic variability (Di Iulio et al., 2019; Lange et al., 2019; Ahles et al., 2010).

Due to relevant disease-related factors such as age of onset, life expectancy, disease progression, and clinical treatments, CRCI has been investigated primarily in patients with breast cancer (Wefel et al., 2015). Only a few studies have been carried out in different adult cancer populations, such as patients suffering from non-small-cell (NSCLC) and small-cell (SCLC) lung cancer (Wefel et al., 2015; Joly et al., 2015), highlighting the presence of cognitive dysfunctions. Lung cancer is one of the most prevalent malignancies worldwide and remains the leading cause of cancer-related mortality (Lane and Smith, 2023). Among lung cancer patients diagnosed at an early stage, surgical intervention is considered the most effective treatment option, offering the best chances of survival (Paoletti et al., 2011). However, for individuals who are not candidates for surgery, treatment primarily relies on chemotherapy, immunotherapy, or a combination of these approaches (Barta et al., 2019). Moreover, the aggressive nature of lung cancer often results in rapid disease progression, which may further contribute to cognitive decline in affected individuals (Mohammed et al., 2011). Indeed, lung cancer survivors may be particularly vulnerable to cognitive decline. A meta-analysis of 12 studies reported that up to 26% of lung cancer survivors experience CRCI, a prevalence comparable to that observed in breast cancer survivors (15%−25%) (Ho et al., 2024). Lung cancer patients may also experience neurological complications due to treatment-related side effects, which may further impair cognitive function and create difficulties in managing daily activities (Giglio and Gilbert, 2010). Emerging evidence suggests that cognitive dysfunction may be a critical factor influencing both QoL and long-term outcomes in lung cancer survivors (Simó et al., 2015).

A study conducted on SCLC patients undergoing chemotherapy found notable declines in visuospatial and verbal fluency abilities (Simó et al., 2015). Another research on NSCLC patients has found marked cognitive decline 1 month after the chemotherapy, with relative improvement at 7-month follow-up (Whitney et al., 2008). In addition, it was seen that cognitive deficits in those patients can also occur independently from the treatment used (Simó et al., 2015).

In addition, psychological factors also play a role in the development of CRCI in lung cancer patients (Hou et al., 2024). Among these, anxiety emerges as the most impactful, being the factor most frequently linked to CRCI in lung cancer patients (Eggen et al., 2020; Kang et al., 2019; Takemura et al., 2022). Depression and sleep disorders have also been identified as potential risk factors for the development of cognitive impairment in this population (Kang et al., 2019; Duivon et al., 2022).

Furthermore, lifestyle factors, such as smoking, have been associated with CRCI (Hou et al., 2024). Research suggests that reduced daily physical activity, along with smoking behaviors, may contribute to a heightened risk of perceived cognitive decline (Hou et al., 2024).

The main objective of this exploratory study is to explore the occurrence of cancer and adjuvant therapy-related cognitive impairment in patients with both NSCLC and SCLC. The primary endpoint measure will be the presence of cognitive impairment during the first year after enrollment through the administration of a comprehensive neuropsychological assessment. In addition, this study will assess the impact of various risk factors on the cognitive profile of lung cancer patients, including psychological symptoms (anxiety, depression, sleep disorders), cognitive reserve, and smoking's potential influence on cognitive dysfunction onset. It will also investigate distinct trajectories of cognitive functioning within the first-year post-treatment across various categories: tumor type (NSCLC vs. SCLC) and chosen adjuvant treatment (none, chemotherapy, immunotherapy, combined chemotherapy, and immunotherapy).

## 2 Methods

### 2.1 Ethical approval and trial registration

This study is a longitudinal trial involving lung cancer patients who are being treated at the European Institute of Oncology (IEO). Ethical approval for the study was obtained from the Ethical Committee of the Institutes of Scientific Research and Healthcare (IRCCS) at the IEO in December 2023 (UID_IEO 2027). The study has also been registered on ClinicalTrials.gov (Identifier: NCT06727370).

### 2.2 Inclusion and exclusion criteria

Patients with a confirmed diagnosis of SCLC or NSCLC (stage I–IV) who are at least 18 years old and can speak and read fluent Italian will be considered eligible. Eligible participants must have provided written informed consent after receiving a detailed explanation of the study procedures. Specifically, both SCLC and NSCLC patients who are candidates for lung surgery or patients who will undergo adjuvant therapy treatment (i.e., chemotherapy or immunotherapy) will be recruited.

Exclusion criteria include the presence of brain metastases, a history of lung cancer, or any concomitant neurological or psychiatric disorder. Patients undergoing brain radiotherapy or those over the age of 70 will also be excluded from the study. The inclusion and exclusion criteria are summarized in Table 1.

**Table 1:** Eligibility criteria.

**Inclusion criteria**	**Exclusion criteria**
• Small-cell and non-small-cell lung cancer (stage I, II, III, IV)	• Presence of brain metastases
• Age ≥ 18 years	• Age ≥ 70
• Patients able to speak and read the local language(s) fluently	• Patients with concomitant neurological or psychiatric disorders
• Having signed the informed consent form, after a detailed explanation of the task and the tools used in the study	• Patients undergoing brain radiotherapy
• Patients who are candidates for lung surgery	• Previous diagnosis of lung cancer
• Patients who will undergo adjuvant treatment (i.e., chemotherapy or immunotherapy)	

### 2.3 Procedure and study design

During the screening visit at the IRCCS IEO, patients who meet the eligibility criteria will be identified by the physicians.

This monocentric study is conducted by the Psycho-Oncology Division of the IEO in collaboration with the Thoracic Surgery Division. Patient selection is carried out by the medical team during multidisciplinary meetings, based on predefined inclusion and exclusion criteria. Eligible patients are then approached by a psychologist, who provides them with detailed information about the study. The characteristics of the study will be accurately exposed and patients will be invited to participate by signing a written informed consent. Participation in the study is voluntary and without charge. Upon obtaining informed consent, a specialized neuropsychologist conducts a comprehensive neuropsychological and psychological assessment. The assessments take place within the hospital in a dedicated setting for cognitive testing, ensuring a controlled and standardized evaluation environment. All methodologies outlined in the study protocol will adhere to relevant guidelines and regulations. Relevant patients' clinical factors, such as age, educational level, and concomitant medications will be collected on the case report forms.

### 2.4 Timing of assessment

The neuropsychological and psychosocial assessment procedures will include three-time points: a baseline assessment (T0), a follow-up at 4 months after the end of treatment (T1), and a second follow-up at 12 months after the end of treatment (T2), as shown in Table 2. T0 will be conducted before the initiation of any cancer-specific treatment to capture baseline cognitive and psychological functioning. The 'end of treatment' is defined as the completion of adjuvant therapy or the primary oncological intervention outlined in the treatment protocol. The time interval between T0 and T1 may vary based on individual treatment plans; however, these variations will be considered in the analysis phase to account for potential confounding factors.

**Table 2:** Neuropsychological and psychosocial timing of assessment.

**Psychological assessment**	**Baseline (T0)**	**4-months follow-up (T1)**	**12-months follow up (T2)**
**Psychosocial assessment**			
FACT-cog	X	X	X
CRIq	X	–	–
BDI-II	X	X	X
STAI	X	X	X
PSQI	X	X	X
EORTC-QOL	X	X	X
**Neuropsychological assessment**			
MOCA	X	X	X
MMSE	X	X	X
FAB	X	X	X
RAVLT	X	X	X
TMT	X	X	X
Digit Span Tests	X	X	X
Corsi Block-tapping Test	X	X	X
SDMT	X	X	X
SCWT	X	X	X
VFT	X	X	X

FACT-cog, Functional Assessment of Cancer Therapy-Cognition; BDI-II, Beck Depression Inventory-II; STAI, State-Trait Anxiety Inventory; EORTC-QoL-C30, EORTC-Quality of Life of cancer patient; PSQI, Pittsburgh Sleep Quality Index; MOCA, Montreal Cognitive Assessment; MMSE, Mini-Mental State Examination; FAB, Frontal Assessment Battery; RAVLT, Ray Auditory Verbal Learning Test; TMT, Trail Making Test; SCWT, Stroop Test Color and Word Test; SDMT, Symbol Digit Modalities Test; VFT, Verbal fluency test; CRIq, Cognitive Reserve Index questionnaire.

### 2.5 Neuropsychological assessment

A neuropsychologist will administer to each lung cancer patient a complete cognitive evaluation at different time points (Table 3). Specifically, tests will be administered to investigate overall cognitive functioning, executive functions, learning and memory, working memory, and attention. Where parallel test forms are available, they will be used at follow-ups to account for learning effects. The comprehensive neuropsychological assessment lasts approximately one hour and a half and includes both the administration of neuropsychological tests and self-report psychological questionnaires, which are completed by patients on-site during the same session. The list of specific tests used in the cognitive assessment can be found in Table 3.

**Table 3:** List of tests used for cognitive assessment.

**General domain**	**Specific domains**	**Neuropsychological tools**
Global cognitive functioning	Memory, attention, executive functions, language, visuospatial abilities, orientation	Montreal Cognitive Assessment
	Orientation, memory, attention, language, visuospatial abilities	Mini-Mental State Examination
Executive functions	Inhibition, cognitive flexibility, planning, action control, interference sensitivity	Frontal Assessment battery
	Inhibitory control	Stroop Color and Word Test
	Lexical access, cognitive flexibility	Verbal Fluency Test
Memory	Verbal episodic memory	Ray Auditory Verbal Learning Test
	Working memory, short-term memory	Digit Span Test
	Visuospatial memory	Corsi Block-tapping Test
Attention/processing speed	Divided visuo-spatial attention, cognitive flexibility	Trail Making Test
	Processing speed, sustained and divided attention	Symbol Digit Modalities Test

#### 2.5.1 Global cognitive functioning

Since many neuropsychological tests do not have specific validity for cancer patients, we will administer two different screening tests, the Mini-Mental State Examination (MMSE; Folstein et al., 1975) and the Montreal Cognitive Assessment (MoCA), to identify which one has greater sensitivity in detecting cognitive deficits in this clinical population. In particular, the MMSE is a rapid (i.e., between 5 and 10 min) neurocognitive screening tool widely used to identify cognitive deterioration (Folstein et al., 1975; Measso et al., 1993). It contains 11 tasks assessing orientation, memory, attention, ability to respond to verbal and written commands, and copy of a figure (Pangman et al., 2000). The raw score will be corrected using the normative data published in the study by Measso and collaborators (Measso et al., 1993).

Instead, the MOCA is a 10-min cognitive screening battery targeted at detecting mild cognitive impairment (Nasreddine et al., 2005). It covers eight cognitive domains: short-term and delayed verbal memory, visuospatial abilities, executive functions, attention, concentration, working memory, language, and orientation to time and place (Nasreddine et al., 2005; Santangelo et al., 2015). The raw score will be corrected using the normative data published in the study by Santangelo and collaborators (Santangelo et al., 2015).

#### 2.5.2 Executive functions

For the general evaluation of executive functions will be used the Frontal Assessment Battery (FAB; Dubois et al., 2000; Aiello et al., 2022). It is a global executive function screening battery that takes about 10 min to be administered. It consists of six subtests that examine cognitive performances related to the frontal lobes: conceptualization, mental flexibility, motor programming, sensitivity to interference, inhibitory control, and environmental autonomy (Lima et al., 2008). The raw score will be corrected using the normative data published in the study by Aiello and collaborators (Aiello et al., 2022).

In addition to this screening battery, some specific tests will be administered to analyze the executive functions of lung cancer patients. The Stroop Color and Word Test (SCWT; Stroop, 1935; Brugnolo et al., 2016) is a cognitive tool that assesses selective and sustained attention and inhibition mechanisms. It is composed of three pages, showing colored words, colored circles, and words colored with incongruent ink; patients must read the three pages as fast as possible. The raw score will be corrected using the normative data published in the study by Brugnolo and collaborators (Brugnolo et al., 2016).

Finally, the Verbal Fluency Test (VFT; Benton et al., 1989; Costa et al., 2014) is used to investigate lexical skills and semantic knowledge. It will be administered to explore mental flexibility and the ability to inhibit intrusive responses. Patients must pronounce as many words as possible beginning with the first letter in 60 s. The raw score will be corrected using the normative data published in the study by Costa and collaborators (Costa et al., 2014).

#### 2.5.3 Memory

The Ray Auditory Verbal Learning Test (RAVL; Rey, 1958; Carlesimo et al., 1996) will be administered to evaluate learning and verbal memory. A list of words is read to the patient who will repeat all the memorized words, this process will be repeated five times. After a delay of 15 min, the patient will list all memorized words without suggestion. This test provides two scores: an immediate memory score, based on the repetition of words across the five trials, and a delayed memory score, based on recall after 15 min. The raw score will be corrected using the normative data published in the study by Carlesimo and collaborators (Carlesimo et al., 1996).

The verbal short-term memory will be assessed using the forward and backward Digit Span test (Wechsler, 1940; Monaco et al., 2013). Sequences of digits will be presented to the patients asking to reproduce them immediately after the presentation. Instead, The Corsi Block-tapping test (Monaco et al., 2013; Corsi, 1972) will be administered to assess visuospatial short-term memory. Sequences of block positions will be presented to the patients asking to reproduce them immediately after the presentation. The raw score of both Digit Span test and Corsi Block-tapping test will be corrected using the normative data published in the study by Monaco and collaborators (Monaco et al., 2013).

#### 2.5.4 Attention

The Trail Making Test (TMT; Reitan, 1956; Siciliano et al., 2019) will be used to assess psychomotor speed and attentional set-shifting. The TMT is composed of two parts: in part A, the patients will connect 25 circled numbers in the correct serial order as quickly as possible, in part B, they will alternately connect circled numbers and circled letters as quickly as possible. The raw score will be corrected using the normative data published in the study by Siciliano and colleagues (Siciliano et al., 2019).

Attention and information processing speed will be evaluated using the Symbol Digit Modalities Test (SDMT; Smith, 1982; Nocentini et al., 2006). The patients will make as many associations as possible between symbols and numbers within 90 s. The raw score will be corrected using the normative data published in the study by Nocentini and collaborators (Nocentini et al., 2006).

### 2.6 Psychosocial assessment

All participants in the study will be required to fill out a set of self-report questionnaires aimed at assessing psychosocial variables. Additionally, the cognitive reserve of each patient will be assessed through the Cognitive Reserve Index questionnaire (CRIq; Nucci et al., 2012). All tests are administered during the visit, and patients complete the self-report questionnaires on-site within the same session. Table 4 outlines the specific questionnaires used to explore these psychological aspects.

**Table 4:** List of self-report questionnaires used to investigate psychological variables and a test for cognitive reserve.

**Domain**	**Questionnaires**
Perceived cognitive impairment	Functional Assessment of Cancer Therapy-Cognition
Depression	Beck Depression Inventory-II
Anxiety	State-Trait Anxiety Inventory-Y form
Quality of sleep	Pittsburgh Sleep Quality Index
Quality of life	EORTC-Core Quality of Life of cancer patient
Cognitive reserve	Cognitive Reserve Index Questionnaire

#### 2.6.1 Self-perceived cognitive impairment

The Functional Assessment of Cancer Therapy-cognition (FACT-COG) will be administered to assess subjective cognitive deficits in cancer patients (Bonomi et al., 1996; Joly et al., 2012). It is a self-report questionnaire composed of 37 items divided into five subscales: perceived cognitive impairments, impact of perceived cognitive impairments on QoL, comments from others, and perceived cognitive abilities.

#### 2.6.2 Depression and anxiety

To assess depressive and anxious symptoms, two different self-report questionnaires, the Beck Depression Inventory-II (BDI-II) and the State-Trait Anxiety Inventory-Y form (STAI-Y) will be assessed. The BDI-II (Beck et al., 1996; Ghisi et al., 2006) is composed of 21 items examining the presence and severity of depressive symptomatology, whereas the STAI-Y (Spielberger et al., 1983; Ilardi et al., 2021) is composed of 40 items, 20 items for trait anxiety and 20 items for state anxiety, respectively.

#### 2.6.3 Quality of sleep

The Pittsburgh Sleep Quality Index (PSQI; Buysse et al., 1988; Curcio et al., 2013) will be administered to investigate sleep quality and disturbances in sleep patterns. It is a self-report questionnaire composed of 19 items generating seven component scores: sleep latency, sleep duration, subjective sleep quality, habitual sleep efficiency, sleep disturbances, use of sleeping medication, and daytime dysfunction. The raw score will be corrected using the normative data published in the study by Curcio and collaborators (Curcio et al., 2013).

#### 2.6.4 Quality of life

The EORTC-Core Quality of Life of cancer patients (EORTC-QoL-C30; Aaronson et al., 1993; Pilz et al., 2022) is a 30-item instrument that will be administered to measure the quality of life in the oncological population.

### 2.7 Sample size

Based on IEO's clinical experience, we project an enrollment of ~200 patients.

Since there is uncertainty regarding the true proportion in the study population of patients developing cognitive impairment during the first year after enrollment, for conservative purposes, we assume a 50% proportion. This assumption represents the worst-case scenario in terms of precision, as it corresponds to the maximum variance in the estimated proportion.

Under this assumption, and with the projected 200 patients, we expect to estimate the proportion of cognitive impairment during the first post-enrollment year with a precision of ±7%, as measured by the width of the 95% confidence interval.

### 2.8 Statistical analyses

The primary endpoint will measure the occurrence of cognitive impairment during the year post-enrollment, treated as a binary outcome. If a patient manifests signs of cognitive impairment at any point during the follow-up or at baseline, based on criteria outlined in the primary objective section, it will be categorized as cognitively impaired. The proportion of cognitively impaired patients will be computed using the total enrolled participants as the denominator. The 95% confidence intervals for this proportion will be calculated using the normal approximation method.

Differences in proportions across strata defined by clinical and treatment variables will be investigated using univariable logistic regression models, considering the presence of cognitive impairment as the dependent variable, and the variable of interest as the independent variable. A *p*-value below 0.05, associated with the parameter estimate of the effect of the independent variable on the outcome, will indicate a potential difference among the strata. Multiple logistic regression models will also be used to control for potential confounders when estimating the parameters of interest.

To assess changes in cognitive function trajectories during the first year post-treatment among distinct strata (e.g., tumor type: NSCLC vs. SCLC, and selected adjuvant treatment: none, chemotherapy, immunotherapy, or combined chemotherapy and immunotherapy), generalized linear mixed models for repeated measures will be used, applied both to binary outcomes (i.e., presence or absence of cognitive impairment at the time of psychological assessment) or continuous outcomes (i.e., the composite cognitive score and the individual scores from the various tests used to derive the composite cognitive score, at the time of psychological assessment).

Specifically, to assess cognitive performance as a continuous measure across different tests and domains, t-scores will be obtained from each neuropsychological test using the mean and SD of normative data of the healthy Italian population. Finally, the mean of t scores will be calculated to generate an overall composite cognitive score. Interaction terms between the time of psychological assessment and the covariate of interest (e.g., tumor type) will be included in the model. A *p*-value below 0.05 related to the interaction will hint at the possibility of the covariate influencing the trajectory of the response variable of interest.

Cognitive impairment will be classified following the International Cognition and Cancer Task Force (ICCTF) recommendations (Wefel et al., 2011), which advocate for a standardized, multi-test approach rather than relying solely on a single cognitive screening tool.

Patients will be classified as cognitively impaired if they meet one of the following criteria:

- Two or more test scores ≤ −1.5 SDs from the normative mean, or
- One test score ≤ −2.0 SDs from the normative mean.

The proportion of patients classified as cognitively impaired will be estimated, with 95% confidence intervals calculated using the normal approximation method.

Differences in the proportion of cognitively impaired patients across strata defined by clinical and treatment variables will be examined using univariate and multivariate logistic regression models, where cognitive impairment (yes/no) will be the dependent variable, and the variables of interest will be the independent variables. A *p*-value < 0.05 associated with the parameter estimate of the independent variable will indicate a potential difference between strata. Adopting the criteria proposed by the ICCTF, cognitive impairment will be identified as two or more test scores at or below −1.5 standard deviations (SDs) from the reference cohort mean, or a single test score at or below −2.0 SDs from the reference cohort mean. The respective reference cohort for each test corresponded to a healthy Italian normative control group provided for each cognitive test. This approach is in line with the methodology outlined by Bartels and collaborators (Bartels et al., 2021). This approach is in line with the methodology outlined by Bartels and collaborators (Bartels et al., 2021).

Changes in cognitive function trajectories during the first-year post-treatment across different strata (e.g., tumor type: NSCLC vs. SCLC and treatment type: none, chemotherapy, immunotherapy, or combined chemotherapy and immunotherapy) will be assessed using generalized linear mixed models (GLMMs) for repeated measures. These models will be applied to both:

- Binary outcomes—presence or absence of cognitive impairment at the time of psychological assessment.
- Continuous outcomes—individual scores from the neuropsychological tests used to derive the cognitive impairment status.

To analyze cognitive performance as a continuous measure, t-scores will be derived from each neuropsychological test using the mean and SD of normative data from the healthy Italian population. A composite cognitive score will then be computed as the mean of the t-scores from the selected neuropsychological tests.

To evaluate potential differences in cognitive trajectories across patient subgroups, interaction terms between time of psychological assessment and the covariate of interest (e.g., tumor type) will be included in the model. A *p*-value < 0.05 associated with the interaction term will suggest that the covariate may influence cognitive changes over time.

In addition to the neuropsychological assessment used to define cognitive impairment, several self-report questionnaires will be analyzed to examine their trajectories over time and their associations with cognitive impairment.

These variables will be analyzed longitudinally at baseline, 4-month, and 12-month assessments. Generalized linear mixed models (GLMMs) for repeated measures will be used to assess changes over time and to determine potential differences between cognitively impaired and non-impaired patients.

Finally, where relevant, multivariate regression models will include sex as an explanatory variable to explore its potential role in modulating the neuropsychological and psychological outcomes analyzed.

### 2.9 Data management and availability

This study involves the collection and processing of personal data, which will be handled in compliance with Regulation (EU) 2016/679 (General Data Protection Regulation—GDPR). Confidentiality, privacy, and data protection measures have been implemented to ensure that all information remains secure and anonymized.

Personal and sensitive data, will be processed using a coding system, whereby each participant is assigned a unique identification code. This ensures that data remains untraceable to individuals, except when necessary for research purposes. Only the study researcher and authorized personnel will have access to the key linking participant identities to their coded data.

All collected data will be stored in secure Excel databases and used to analyze participants' perceptions of the provided treatments. The findings will be disseminated through scientific publications and conference presentations, ensuring that only anonymized and aggregated data are shared.

Data will be securely stored in protected databases at the IEO for up to 10 years after study completion, with retention periods subject to local regulations.

## 3 Discussion

The purpose of this study is to investigate the insurgence of CRCI in patients diagnosed with both NSCLC and SCLC through neuropsychological assessment within the first year after enrollment. Referring to the timing of assessment in the literature (Schroyen et al., 2022), the adoption of three different time points allows us to have continuous monitoring of the course of cognitive performance in lung cancer patients. The pre-treatment assessment (T0) allows a focus on the effect of the disease on cognition before drug treatment. An intermediate assessment (T1), on the other hand, allows an in-depth study of the acute effect of treatments on cognitive functions before compensatory processes take over. Finally, a long-term assessment (T2) makes it possible to investigate the spontaneous recovery implemented to counter the neurotoxic effect of certain cancer treatments (Di Iulio et al., 2019).

Indeed, several studies have shown that the onset of CRCI is multifactorial, thus being influenced mainly by carcinoma-related inflammatory processes and pharmacological treatments (Lange et al., 2019; Pendergrass et al., 2018; Oppegaard et al., 2023). Particularly, treatments like chemotherapies, due to the related neurotoxicity, are associated with the release of pro-inflammatory cytokines that contribute to the onset of clinical difficulties (Di Iulio et al., 2019).

Therefore, the manifestation of cognitive deficits is variable in terms of both severity and timing of onset. In this vein, it becomes essential to have a neuropsychological battery that would be sensitive to the dysfunctions of cancer patients and enable the early and timely detection of the slightest changes in cognitive functioning.

Furthermore, patients with lung cancer are particularly susceptible to the onset of cognitive deficits. In a recent study, Bartels et al. (2021) observed that over one-third of lung cancer patients exhibited neuronal autoantibodies that were found to be associated with cognitive impairment (Bartels et al., 2021). Particularly in SCLSC patients, the likelihood of cognitive impairment was 11 times higher compared to patients without autoantibodies (Bartels et al., 2021), suggesting that neuronal autoantibodies might play a pathogenic role in CRCI among lung cancer patients.

Moreover, this study aims to set up a comprehensive assessment, giving emphasis also on the patient's subjective experiences of cognitive difficulties. This would make it possible both to detect variations in performance that are difficult to detect by objective neuropsychological tests and to involve patients in their care pathway (Hutchinson et al., 2012; Biglia et al., 2012; Országhová et al., 2021). This mechanism would also foster an active exchange with healthcare professionals and facilitate the decision regarding the need for further psychological intervention (Mayo et al., 2021).

To this end, within our study, the influence of various individual and risk factors on the cognitive profile of lung cancer patients will also be analyzed: the identification and understanding of risk factors associated with cognitive dysfunction in patients with lung cancer may have significant implications for clinical practice (Lange et al., 2019; Mayo et al., 2021). Clinicians may be able to identify patients at risk of developing cognitive dysfunction through an assessment of risk factors such as psychological symptoms, cognitive reserve, and tobacco use. This would allow the implementation of targeted preventive interventions to reduce the risk of cognitive decline and improve the quality of life for patients (Országhová et al., 2021; Bai and Yu, 2021).

Furthermore, longitudinal assessment of cognitive function during treatment and follow-up may provide valuable insights into the progression of cognitive decline over time and the effectiveness of various treatments in mitigating or slowing down this progression. This could guide clinical decisions regarding the management and monitoring of cognitive function in patients with lung cancer, allowing for more targeted and personalized patient management (Gorini and Pravettoni, 2011; Gorini et al., 2018). However, it is important to note that although the ICCTF recommends longitudinal studies with repeated assessments to evaluate changes in cognitive function (Wefel et al., 2011), some modifications may be caused by the repetition of the same tests (Bartels et al., 2010). For this reason, it is advisable to use parallel versions of the tests when available.

Moreover, psychological reactions to a cancer diagnosis, such as anxiety, depression, and distress, are common and may contribute to cognitive impairment even before the start of cancer treatment (Kaiser et al., 2019). Previous research has shown the association between cognitive difficulties and psychological factors, including anxiety, depression, post-traumatic stress symptoms and negative affect (Dhillon et al., 2018; Pullens et al., 2013; Seliktar et al., 2015; Danhauer et al., 2013). In addition, fatigue, often reported by cancer patients, has been linked to cognitive impairment and may act as a confounding factor in neuropsychological assessments (Dhillon et al., 2018; Pullens et al., 2013; Seliktar et al., 2015; Danhauer et al., 2013). To account for these influences, our study includes a baseline assessment conducted before the start of cancer-specific treatments, which allows us to examine the impact of preexisting psychological distress on cognitive outcomes. In addition, we will consider key factors such as educational level and cognitive reserve, which have been identified as potential moderators of cognitive performance.

In addition to the most analyzed confounding factors such as subject age, gender, and education, this study aims to assess the impact of various risk factors on cognitive function, including psychological symptoms, cognitive reserve, and smoking. However, there may be additional confounding variables not accounted for in the analysis, which could influence the observed associations between variables. To this end, future research should gather extensive data on potential confounding variables, such as socioeconomic status, education level, physical health conditions, medication use, and lifestyle factors. This comprehensive approach allows for the identification and adjustment of a wider range of confounders, thereby improving the accuracy of the observed associations.

Finally, the development and validation of a battery of neuropsychological tests specifically tailored to lung cancer patients could facilitate accurate assessment of cognitive function in this population, detecting even minimal changes in cognitive function and allowing for the introduction of personalized cognitive rehabilitation plans (Capetti et al., 2024).

### 3.1 Study limitations

Despite the fundamental contributions of this study in the field of possible neuropsychological consequences in SCLC and NSCLC cancer patients, there are several potential limitations to consider in the study. Firstly, the generalizability of the findings may be limited due to the focus on patients from a specific institution: the study will be conducted in one center, thus reducing the accessibility to greater variability of subjective experiences. To mitigate this aspect, the multicenter collection could be implemented to obtain a more heterogeneous and representative sample. In addition, a comparative analysis with data from similar studies conducted in other centers could be useful to check the consistency of the results.

Additionally, there is the possibility of measurement bias in the assessment of cognitive impairment. While a comprehensive neuropsychological assessment will be used, factors such as patient motivation and fatigue could influence cognitive test performance, potentially biasing the results. In addition, the absence of a control group in the experimental design may represent an additional limitation of the study.

To address potential measurement bias, assessment sessions could be scheduled to ensure the best conditions for patients to perform the cognitive assessment. Selecting times of the day when patients are most active, limiting the duration of assessment, and including regular breaks could be effective strategies to reduce the impact of fatigue. Given the possible limited resources available to patients, we will prioritize the most important tests and shorten to assessment when necessary, such as allowing self-report tests to be completed remotely. Each cognitive test will be performed balancing accuracy with the need to minimize the burden on patients. Flexibility in scheduling assessments will also be maintained, allowing adjustments in response to individual patient needs, such as physical or emotional factors that might affect their performance. These strategies aim to improve data accuracy while respecting the wellbeing of participants.

Another possible limitation of the study is the repetition of fluency tasks. To reduce this risk, we use parallel versions of the tests whenever possible and, in cases of letter overlap between tasks, calculate the final fluency test score by summing the words produced for each letter across the various tests.

Moreover, the neuropsychological tests adopted are validated on different clinical populations and may not be sensitive enough to detect subtle cognitive changes in cancer patients who do not have severe deterioration (Howieson, 2019). Indeed, although existing guidelines recommend the use of certain neuropsychological tests for cancer patients (Wefel et al., 2011; Lange et al., 2019; Mayo et al., 2021), there is currently no disease-specific normative data or comprehensive test batteries designed to assess the full cognitive profile of lung cancer patients. Future studies should validate cognitive assessment tools specific for SCLC and NSCLC cancer patients, which can investigate the cognitive alterations characterizing these patients with high accuracy. Finally, another limitation of the study is related to the availability of normative data for younger patients (aged 18–35 years). In many cases, the normative data used for test score corrections are not sufficiently representative of this age group. To address this issue, for patients in this age range, we will apply the correction formula provided in the reference papers, which has been used to derive normative data for this population.

## 4 Conclusion

Overall, the findings from this longitudinal study will contribute to a deeper understanding of cognitive impairment in NSCLC and SCLC lung cancer patients, potentially informing clinical practice and paving the way for the development of targeted interventions to improve patient outcomes and quality of life. The examination of cognitive effects related to lung cancer and its clinical treatments seems to be able to provide important information regarding the clinical management of this oncology population. However, further research will be essential to validate and expand upon these initial findings.
